# Secretory PLA_2_-IIA: a new inflammatory factor for Alzheimer's disease

**DOI:** 10.1186/1742-2094-3-28

**Published:** 2006-10-07

**Authors:** Guna SD Moses, Michael D Jensen, Lih-Fen Lue, Douglas G Walker, Albert Y Sun, Agnes Simonyi, Grace Y Sun

**Affiliations:** 1 Laboratory of Neuroinflammation, Sun Health Research Institute, Sun City, AZ 85372, USA; 2 Biochemistry Department, University of Missouri-Columbia, Columbia, MO 65211, USA; 3 Department of Medical Pharmacology and Physiology, University of Missouri-Columbia, Columbia, MO 65211, USA

## Abstract

Secretory phospholipase A_2_-IIA (sPLA_2_-IIA) is an inflammatory protein known to play a role in the pathogenesis of many inflammatory diseases. Although this enzyme has also been implicated in the pathogenesis of neurodegenerative diseases, there has not been a direct demonstration of its expression in diseased human brain. In this study, we show that sPLA_2_-IIA mRNA is up-regulated in Alzheimer's disease (AD) brains as compared to non-demented elderly brains (ND). We also report a higher percentage of sPLA_2_-IIA-immunoreactive astrocytes present in AD hippocampus and inferior temporal gyrus (ITG). In ITG, the majority of sPLA_2_-IIA-positive astrocytes were associated with amyloid β (Aβ)-containing plaques. Studies with human astrocytes in culture demonstrated the ability of oligomeric Aβ_1–42 _and interleukin-1β (IL-1β) to induce sPLA_2_-IIA mRNA expression, indicating that this gene is among those induced by inflammatory cytokines. Since exogenous sPLA_2_-IIA has been shown to cause neuronal injury, understanding the mechanism(s) and physiological consequences of sPLA_2_-IIA upregulation in AD brain may facilitate the development of novel therapeutic strategies to inhibit the inflammatory responses and to retard the progression of the disease.

## Background

Alzheimer's disease (AD) is the most prevalent neurodegenerative disease affecting the aging population, and is characterized by memory loss and decline in cognitive functions. Some of the characteristic landmarks of the disease include neurofibrillary tangles [1] and amyloid plaques, which are frequently surrounded by reactive astrocytes and activated microglial cells as well as dystrophic neurites [2,3]. The presence of activated glial cells and the increase in inflammation-associated proteins in AD brain support the neuroinflammatory nature of this disease [4-9]. Increased amounts or deposits of inflammatory proteins such as the classical and alternative complement proteins and acute phase reactant proteins have been reported in AD brains, as have increased microglial expression of the major histocompatibility complex (MHC) antigens [10]. Although the underlying mechanism(s) for neuroinflammation in AD brain is not clearly understood, there is considerable evidence supporting a role for specific forms of amyloid beta peptide (Aβ) in inducing production of pro-inflammatory cytokines by microglia and astrocytes [5,11-13]. Therefore, understanding the mechanisms that modulate neuroinflammatory responses and their impact on neuronal degenerative processes may help to uncover important elements of the disease and to develop new treatment strategies [14-16].

The phospholipases A_2 _(PLA_2_) belong to a family of enzymes that are widely expressed in many types of mammalian cells [17]. These enzymes not only play a role in maintenance of cell membrane phospholipids, but are also actively involved in the production of arachidonic acid (AA), the precursor for prostanoids [18,19]. Among more than 20 different forms of PLA_2 _identified, there is considerable attention on the group IV calcium-dependent cytosolic PLA_2 _(cPLA_2_) and the group II secretory PLA_2 _(sPLA_2_). Both groups of PLA_2 _can participate in the oxidative and inflammatory responses in neurodegenerative diseases [20-25]. Although previous studies have demonstrated an increase in mRNA expression [26] and immunoreactivity of cPLA_2 _in AD brains [26-28], studies to relate sPLA_2_-IIA expression with AD have been lacking. In the periphery, sPLA_2_-IIA is regarded as an inflammatory protein, and is involved in inflammatory diseases such as arthritis, atherosclerosis, acute lung injury, sepsis and cancer [25,29-32]. Secretory sPLA_2_-IIA cannot be studied in transgenic mouse models of AD due to a frameshift mutation of this gene in many mouse strains [33]. However, studies with rat models of brain injury have demonstrated an increase in sPLA_2_-IIA expression associated with different forms of neuronal insults, including cerebral ischemia [34,35] as well as other types of neuronal injuries [36,37].

In this report, we provide data demonstrating up-regulation of sPLA_2_-IIA mRNA and protein expression in reactive astrocytes in AD brains as compared to age-matched non-demented (ND) control brains. In addition, studies with human astrocytes demonstrated the induction of sPLA_2_-IIA mRNA by pro-inflammatory cytokines and Aβ, further supporting an inflammatory role of this enzyme in AD brain.

## Methods

### Human brain tissue

Paraformaldehyde-fixed brain sections for immunohistochemistry were obtained from the Brain Bank of the Sun Health Research Institute (Sun City, AZ). Patients were classified as AD or ND cases by the neuropathological criteria of the Consortium to Establish a Registry for AD (CERAD) and NIA-Reagan guidelines. Postmortem brain samples were obtained from 7 male and 9 female ND subjects and 5 male and 11 female AD subjects (Table 1). The mean age (years) for the AD cases was 86.25 ± 8.22 and for the ND cases was 84.44 ± 6.74 (mean ± SD), and the mean postmortem interval (hours) for AD cases was 2.59 ± 0.45 and for ND cases was 2.63 ± 0.62 (mean ± SD).

**Table 1:** Postmortem human brains used in the study of sPLA_2_-IIA expression

**Cases**	**Clinical Diagnosis**	**Gender**	**Age (years)**	**PMI (hours)**	**Type of Study**	**Brain Region**
1	ND	M	78	2.7	IHC	ITG
2	ND	M	81	2.7	IHC	I TG
3	ND	M	69	2.2	IHC	HPC, ITG
4	ND	M	84	2.5	IHC	HPC, ITG
5	ND	M	78	1.7	IHC	HPC, ITG
6	ND	M	94	3.0	RNA	HPC, CB
7	ND	M	85	3.2	RNA	HPC, CB
8	ND	F	85	2.5	RNA	HPC, CB
9	ND	F	86	2.0	RNA	HPC, CB
10	ND	F	88	3.0	RNA	HPC, CB
11	ND	F	94	2.5	RNA	HPC, CB
12	ND	F	86	2.5	RNA	HPC, CB,
13	ND	F	83	2.5	RNA	HPC, CB
14	ND	F	94	2.3	RNA	HPC, CB,
15	ND	F	78	2.8	IHC	ITG
					RNA	HPC, CB
16	ND	F	88	3.5	RNA	HPC, CB
17	AD	M	86	3.0	IHC	ITG
18	AD	M	87	3.0	IHC	HPC
					RNA	HPC, CB
19	AD	M	79	2.0	IHC	HPC
					RNA	HPC, CB
20	AD	M	94	3.8	IHC	ITG
21	AD	M	92	2.0	IHC	ITG
22	AD	F	89	3.0	IHC	HPC
					RNA	HPC, CB
23	AD	F	80	2.3	IHC	HPC, ITG
24	AD	F	85	1.7	RNA	HPC, CB
25	AD	F	95	3.2	RNA	HPC, CB
26	AD	F	91	3.0	RNA	HPC, CB
27	AD	F	89	2.3	RNA	HPC, CB
28	AD	F	97	1.5	IHC	ITG
29	AD	F	64	3.2	IHC	ITG
30	AD	F	77	2.8	IHC	ITG
31	AD	F	85	2.3	IHC	HPC
32	AD	F	90	3.0	RNA	HPC, CB

Abbreviations: ND: Non Demented Control, AD: Alzheimer's Disease, M: Male; F: Female; PMI: Post Mortem Interval; IHC: Immunohistochemistry; HPC: Hippocampus; ITG: Inferior Temporal Gyrus, CB: Cerebellum.

### Stimulation of sPLA2-IIA mRNA expression in astrocytes from human post-mortem brains

Astrocytes were cultured from superior frontal gyrus of post-mortem brains donated to the Sun Health Research Institute Brain Program according to a protocol described previously [38]. Astrocytes were maintained in Dulbecco's Modified Eagle medium (DMEM) containing 10% fetal bovine serum (FBS).

IL-1β and interferon-γ(IFNγ)(PeproTech, Rocky Hills, NJ) and recombinant Aβ_1–42 _(rPeptide, Bogart, GA) were used to stimulate astrocytes for the study of sPLA_2_-IIA mRNA expression. Lyophilized Aβ_1–42 _were dissolved in 0.1 M NaOH and buffered with phosphate buffered saline to make a final concentration of 500 μM. The peptide solution was subsequently incubated at 37°C for 18 hours to promote oligomerization. Aliquots of the oligomerized Aβ_1–42 _were stored in liquid nitrogen until experiments were performed. Twenty four hours before treatments, culture media was exchanged for serum-free DMEM. Cells were then incubated in serum-free DMEM with IL-1β (20 ng/ml), IFNγ (100 ng/ml), or 2.5 μM Aβ_1–42 _for 24 h at 37°C. After incubation, cells were processed for RNA extraction.

### RNA isolation, reverse transcription polymerase chain reaction (RT-PCR), and real time PCR

RNA was extracted from frozen brains and cultured astrocytes with Trizol reagent according to the manufacturer's instructions (Invitrogen, Carlsbad, CA). RNA was isolated from hippocampus and cerebellum from 10 AD and 10 ND cases (Table 1). The integrity of isolated RNA was confirmed by denaturing agarose gel electrophoresis, and quantified by ultraviolet spectrophotometry. Total cellular RNA (1–2 μg) was reverse transcribed with random hexamers using Superscript III reverse transcriptase (Invitrogen, CA) as previously described [13,39].

RT-PCR was carried out to assess sPLA_2_-IIA mRNA expression in astrocyte cultures. In this study, primers for *sPLA*_2_*-IIA *are: forward 5'- GACTCATGACTGTTGTTACAACC-3' and reverse 5'-TCTCAGGACTCTCTTAGGTACTA-3' that amplify a 493 bp fragment, and primers for *β-actin *are: forward 5'-TGGAGAAGAGCTATGAGCTGCCTG-3' and reverse 5'-GTGCCACCAGACAGCACTGTGTTG-3' that amplify a 289 bp fragment [39]. After amplifications of 40 cycles for sPLA_2_-IIA or 25 cycles for β-actin, a 5 μl aliquot of each reaction mixture was applied to 6% acrylamide gels. Bands were quantified using AlphaEaseFC software (Alpha Innotech, San Leandro, CA). Expression values were normalized for the levels of β-actin, which was used as the reference cellular transcript.

Real time PCR was used for determination of levels of sPLA_2_-IIA mRNA in brain tissues. Taqman primers and probes specific for human sPLA_2_-IIA and ribosomal 18S RNA were obtained from Applied Biosystems (Foster City, CA). For each sample (analyzed in triplicate), a pool containing Brilliant qPCR master mix (Stratagene, La Jolla, CA), Taqman probes, along with the cDNA was prepared, and then aliquoted into 96 well microtiter qPCR plates. Each analysis contained a series of diluted samples for standard curve purposes, as well as negative template and negative reverse transcriptase control samples. The real time PCR was carried out under optimized conditions using a Stratagene Mx3000p qPCR instrument. At the end of the run, relative expression results were calculated from the Ct values of each sample using the Mx3000p operating software. Each run was considered satisfactory if the standard curve covering a 1000-fold dilution range gave R^2 ^of > 0.98. Results were expressed relative to levels of 18S ribosomal RNA present in the samples, which were determined in the same manner.

### Immunohistochemistry

Free-floating 20 μm sections from hippocampus and inferior temporal gyrus (ITG) were cut from 4% paraformaldehyde-fixed human brains and were used to study sPLA_2_-IIA protein expression. Our previously published immunohistochemical procedure was used for this purpose [40]. Sections were sequentially incubated with a monoclonal antibody to sPLA_2_-IIA (Cayman, Ann Arbor, MI; 1:500 dilution, 18 hours, room temperature) in a phosphate buffered saline containing 0.3% Triton-X 100 (PBS-T). This was followed by reaction with biotinylated anti-mouse IgG (Vector Laboratories, Burlingame, CA; 1:2000, 2 hours) and washed with PBS-T before applying avidin-biotin peroxidase complex (ABC) solution (Vector Laboratories, Burlingame CA; 1:2000, 1 hour). We detected bound antibody-antigen enzyme complex by reaction of sections with nickel-enhanced diaminobenzidine (DAB) solution [38,41]. For two-color double immunohistochemistry, brain sections were first immunoreacted with nickel-DAB solution, then washed, and followed by 1% hydrogen peroxide to block peroxidase activity. Subsequently, sections were reacted with a polyclonal antibody to glial fibrillary acidic protein (GFAP; DAKO, Carpinteria, CA) to identify reactive astrocytes. Detection of GFAP was carried out using the same procedure described, with the exception that biotinylated anti-rabbit IgG and DAB substrate without nickel enhancement were used. These procedures produced sPLA_2_-IIA immunoreactivity in dark blue color and GFAP in brown color. In some of the sections, an antibody to amyloid β (3D6, Elan Pharmaceuticals, South San Francisco, CA; 1:2000) was used to detect amyloid plaques. Some of the immunoreacted sections were counterstained with 1% neutral red to provide a general view of the cell populations in tissues. The mounted sections were dehydrated through graded ethanol and coverslipped with Permount embedding solution. The number of sPLA_2_-IIA immunoreactive astrocytes associated with amyloid plaques was counted. Following double immunoreaction with sPLA_2_-IIA and GFAP, sections were mounted and counter-stained with 1% thioflavin S (in 70% alcohol) for 15 minutes, dehydrated in 70% alcohol, and coverslipped with Vectashield mounting medium (Vector Laboratories, CA).

### Quantifying sPLA_2_-IIA-positive astrocytes in AD and ND brain sections

To estimate the percentage of sPLA_2_-IIA-positive astrocytes, we used a semi-quantitative cell counting procedure with brain sections containing dentate gyrus (DG), CA3, or ITG that had been reacted with antibodies to detect sPLA_2_-IIA and GFAP. In each brain region, the total number of GFAP immunoreactive cells and GFAP/sPLA_2_-IIA immunoreactive cells were counted using a 1-mm^2 ^reticle, mounted in the eye-piece of an Olympus microscope, using 20X and 40X objective lenses (Olympus, Melville, NY). In the ITG sections, 10 vertical regions encompassing the width of the 1-mm^2 ^reticle field were counted. In each vertical region, counting began at the outer edge of the molecular layer and finished at the interface of the multiform layer and white matter. Cell counting was performed by a blinded examiner and in each vertical region mean cell numbers from 10 vertical fields were obtained. From this, we calculated the percentage of sPLA_2_-IIA immunoreactive astrocytes in ITG for each case from 6 AD and 6 ND samples. In the CA3 region, we started counting at the CA3 boundary, and counted 5 consecutive, 1-mm^2 ^reticle fields covering the pyramidal cell layers. In the DG region, we began counting at the hilus and counted the 1-mm^2 ^reticle fields consecutively as far as the junction of the DG and CA region. The percentages of sPLA_2_-IIA-positive astrocytes in the DG and CA3 regions were determined from 4 AD and 4 ND cases.

Using the same methodology, the number of sPLA_2_-IIA-positive cells that co-localized with thioflavin S-positive plaques was counted. In each reticle field, thioflavin S-positive plaques were first visualized with a fluorescence microscope followed by phase contrast observation. Percentages of sPLA_2_-IIA-positive astrocytes that co-localized with thioflavin S-positive plaques were obtained from the total number of sPLA_2_-IIA-positive astrocytes.

### Statistical analysis

Student's *t *test, or one-way ANOVA followed by Tukey posthoc multiple comparison test was used to analyze data using the GraphPad Prism 4 software. Significant differences between groups were assumed for P values < 0.05.

## Results

### Expression of sPLA_2_-IIA mRNA in hippocampus and cerebellum of AD and ND brains

To demonstrate sPLA_2_-IIA mRNA expression in human brain, we measured levels of sPLA_2_-IIA mRNA by real time PCR analysis of RNA prepared from hippocampus and cerebellum samples from AD and ND patients. Hippocampal tissues for RNA purification were confined mainly to CA3 and dentate gyrus (DG) areas, as tissues from CA1 were not available. We detected a significant, 4.5-fold increase (p < 0.01) in sPLA_2_-IIA mRNA in AD hippocampus samples as compared to ND. On the other hand, there was no difference between sPLA_2_-IIA mRNA levels in cerebellar samples from AD and ND brains.

### Increased immunoreactivity of sPLA_2_-IIA in astrocytes of AD brain

**Figure 1:**
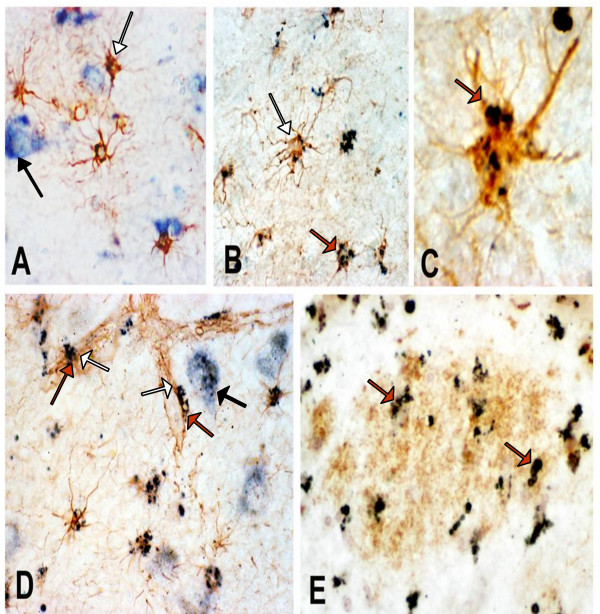
**sPLA_2_-IIA immunoreactivity in human postmortem brain tissues**. Double immunostaining depicting sPLA_2_-IIA immunoreactivity in dark blue color and GFAP immunoreactivity in brown color is shown in panels A-D (using 20X and 40X objective lenses). Panel A demonstrates that little sPLA_2_-IIA immunoreactivity is present in a cluster of GFAP immunoreactive astrocytes in ND hippocampus. Panel B shows many GFAP-positive astrocytes (white arrow) labeled with intense immunoreactivity for sPLA_2_-IIA (dark immunoreactive products, red arrow) in AD hippocampus. At higher magnification (Panel C), sPLA_2_-IIA immunoreactivity is shown in an astrocyte cell body in granular-like structures (red arrow). Panel D shows that immunoreactivity for sPLA_2_-IIA (red arrows) is also present in GFAP-positive astrcoytes (white arrows) surrounding microvessels in AD hippocampus. We also detected sPLA_2 _immunoreactivity in hippocampal neurons (black arrows) in ND (Panel A) and AD (Panel D) hippocampus. In Panel E, several sPLA_2_-IIA immunoreactivitve profiles (red arrows) are co-localized with an amyloid plaque (brown immunoreactive area) detected by immunohistochemistry with an antibody to Aβ.

Immunohistochemistry was used to demonstrate cell-associated sPLA_2_-IIA protein in AD and ND brains. As shown in Figure 1A, there were few GFAP-positive astrocytes present in the hippocampal DG area from ND brain and these cells, which appeared to be forming astrocyte foot contacts with an amyloid plaque, showed little sPLA_2_-IIA immunoreactivity. A higher number of GFAP-positive astrocytes and sPLA_2_-IIA/GFAP-positive astrocytes were present in AD hippocampal regions (Fig. 1B and 1C). Immunoreactivity of sPLA_2_-IIA was also detected in GFAP-positive cells lining the blood vessels (Fig. 1D), and co-localized with amyloid deposits (Fig. 1E).

**Figure 2:**
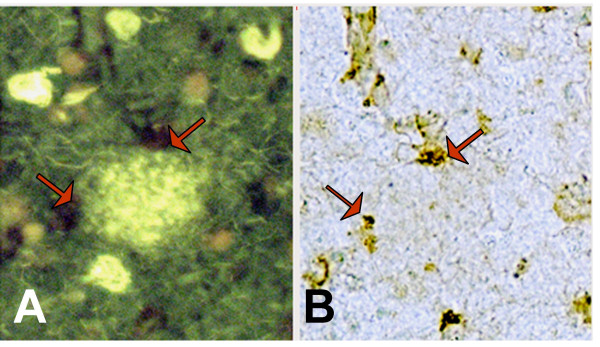
**Co-localization of sPLA_2_-IIA-positive astrocytes with thioflavin S-positive plaques**. Double immunostaining of sPLA_2_-IIA and GFAP combined with thioflavin S staining shows the presence of sPLA_2_-IIA (red arrows) in GFAP-positive astrocytes (panels A and B) and their association with thioflavin S-positive amyloid plaques (green fluorescent area in panel A) in an ITG section from an AD case.

To investigate whether sPLA_2_-IIA-positive astrocytes are co-localized with amyloid deposits that contain Aβ in β-sheet conformation, brain sections double-immunoreacted with sPLA_2_-IIA and GFAP were stained with thioflavin S fluorescence dye. Thioflavin S-positive plaques were present in the DG, CA3, and ITG of all AD cases; no thioflavin S-positive plaques were detected in the DG and CA3 regions of ND cases. Nevertheless, thioflavin S-positive plaques were present in the ITG of two ND cases. A sub-population of sPLA_2_-IIA-positive astrocytes co-localized with thioflavin S-positive plaques in AD patients as demonstrated in the same brain sections that were processed for double immunohistochemistry for GFAP and sPLA_2_-IIA antibodies (Fig. 2B) and for thioflavin S histochemistry (Fig. 2A).

We have quantified the percentages of astrocytes that were immunoreactive for sPLA_2_-IIA and GFAP, and also the percentages of sPLA_2_-IIA-positive astrocytes that are associated with thioflavin S-positive plaques from brain sections containing DG, CA3, and ITG regions in AD and ND patients (see Table 1 for patient information). The results are shown in Table 2. Data show firstly that significantly greater percentages of GFAP-positive astrocytes were immunoreactive for sPLA_2_-IIA in AD cases than in ND cases in all three brain regions. Secondly, in the gray matter of ITG, more than two thirds of sPLA_2_-IIA-positive astrocytes in AD tissue sections co-localized with thioflavin S-positive plaques. Thirdly, among the three brain regions tested, the DG in AD brains contained the highest percentage of sPLA_2_-IIA-positive astrocytes. However, the majority of the sPLA_2_-IIA-positive astrocytes in the hippocampal regions were not associated with thioflavin S-positive plaques.

**Table 2:** sPLA_2_-IIA-positive astrocytes in hippocampus and inferior temporal gyrus of Alzheimer (AD) and nondemented (ND) subjects.

**Brain region**	**Dentate gyrus**		**CA3 region**		**Inferior temporal gyrus**	
**Subjects**	**AD**	**ND**	**AD**	**ND**	**AD**	**ND**

**Total sPLA_2_-IIA-positive astrocytes**	50.82 ± 9.00^1,^***	1.27 ± 0.96	24.11 ± 5.15***	0.00	12.86 ± 2.90***	1.99 ± 0.56
**Plaque-associated sPLA_2_-IIA-positive astrocytes^2^**	0.66 ± 0.21*	0.00	1.59 ± 0.38**	0.00	8.60 ± 2.74*	0.51 ± 0.35

^1^Astrocyte counts are given as percent of all GFAP-positive astrocytes. Values are expressed as mean ± SD.

^2^Plaque-associated astrocytes were identified by co-staining with thioflavin S

*, **, ***Value is significantly different from corresponding ND value (Student's *t *test): *p < 0.01; **p < 0.005; ***p < 0.001

sPLA_2_-IIA immunoreactivity was not detected in microglial cells (not shown); however, sPLA_2_-IIA immunoreactivity was observed in neurons (identified based on their morphology) in both ND and AD brains (Fig. 1A and 1D). Unlike the immunostaining for astrocytes, which showed punctate dark spots, sPLA_2_-IIA immunoreactivity in neurons shows an amorphous distribution pattern.

### Pro-inflammatory cytokines and Aβ*1–42 *induce sPLA_2_-IIA mRNA in human astrocytes

**Figure 3:**
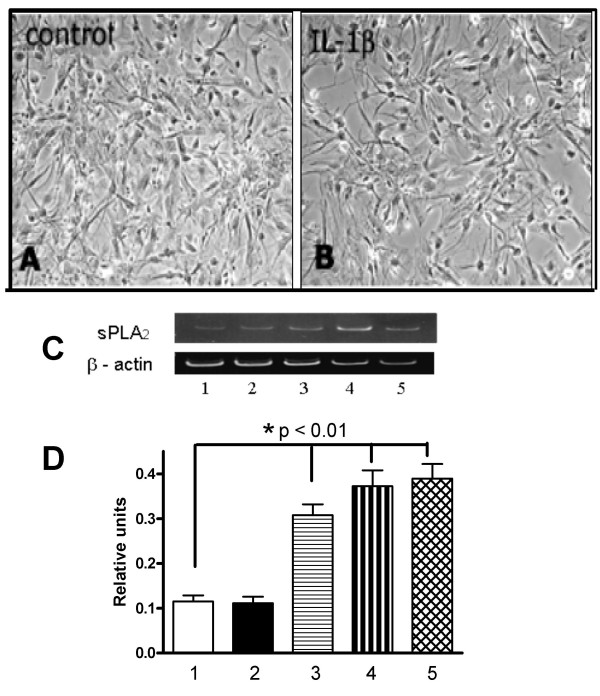
**Induction of sPLA_2_-IIA mRNA expression by cytokines and Aβ _1–42 _in cultured human astrocytes**. Phase contrast micrographs show human astrocytes in control (panel A) and IL-1β-stimulated cultures (panel B) for 24 hours. Human postmortem astrocytes were used for the sPLA2-IIA RNA study. Experiments were performed using cultures derived from 3 neuropathologically confirmed AD cases. A representative gel depicting PCR-amplified fragments for sPLA2-IIA and β-actin is shown in panel C. Gel lanes 1–5 represent the following treatments used in the astrocyte cultures: 1. control; 2. IFNγ (100 ng/ml); 3. Aβ_1–42 _(2.5 μM); 4. IL-1β (20 ng/ml); 5. IL-1β and Aβ_1–42_. Twenty-four hours after treatment, RNA was extracted from cells, reverse transcribed, and RT-PCR was carried out as described in methods. Panel D shows a bar graph depicting relative units of sPLA_2_-IIA expression after normalization with β-actin. Significant differences (*) comparing treatment groups with controls were obtained by one-way ANOVA followed by Tukey multiple comparison post hoc test.

To further demonstrate expression and regulation of sPLA_2_-IIA in astrocytes, human astrocytes cultured from superior frontal gyrus of post-mortem AD brains were treated with Aβ_1–42 _(2.5 μM), IL-1β (20 ng/ml), and IFNγ (100 ng/ml), alone or in combination for 24 hours. When stimulated with IL-1β, astrocytes from AD post-mortem brain developed reactive morphology with slender long processes as compared to untreated astrocytes (Fig. 3A and 3B). RT-PCR indicated very low sPLA_2_-IIA mRNA expression in control and IFNγ -treated astrocytes (Fig. 3C and 3D), but significant increases were observed upon stimulating astrocytes with Aβ_1–42 _and IL-1β. When Aβ_1–42 _and IL-1β were given together, there was no further enhancement of sPLA_2_-IIA mRNA expression, compared to each treatment alone.

## Discussion

In this study, we characterize the expression of sPLA_2_-IIA in AD and ND brains. In AD, severe pathological changes occur, topographically and quantitatively, in the hippocampus and temporal cortical areas, whereas cerebellum is relatively spared from AD pathology. Using real time PCR for measuring sPLA_2_-IIA mRNA in hippocampus and cerebellum, we showed a significant increase in sPLA_2_-IIA mRNA in the hippocampus of AD brains as compared to ND brains, whereas no increase was observed in cerebellum. Using immunohistochemistry, we demonstrated that GFAP-positive astrocytes are the main cell type that express sPLA_2_-IIA protein. In hippocampus and ITG, the percentages of astrocytes that expressed sPLA_2_-IIA protein are significantly higher in the AD brains when compared to ND brains. This is the first demonstration of upregulation of sPLA_2_-IIA protein in astrocytes in AD brains. The increase in sPLA_2_-IIA expression in AD hippocampus, but not in AD cerebellum, is in agreement with the neuropathological observations that reactive astrocytes are increasingly associated with pathology in hippocampus and cortex, whereas diffuse amyloid deposits and limited astrocyte activation are found in cerebellum [3,42].

It has been established that the number of GFAP-positive astrocytes associated with amyloid plaques changes during plaque formation. There are fewer GFAP-positive astrocytes associated with diffuse plaques; while more are associated with neuritic plaques containing fibrillar Aβ and dystrophic neuritis [43]. Thioflavin S fluorescence dye can detect amyloid fibrils in β-pleated sheet formation, a state of aggregation that occurs when diffuse plaques progress to neuritic plaques. Although thioflavin S-positive plaques are more abundant in AD brains, there are occasionally such plaques in the neocortex of normal aging brains [44,45]. In this study, thioflavin S-positive plaques were observed in ITG in 2 ND patients. We analyzed whether increases in the number of sPLA_2_-IIA-positive astrocytes are associated with thioflavin S-positive plaques. Our results indicated that these cells were highly associated with thioflavin S-positive plaques in ITG sections, but not in DG or CA3 regions of the hippocampus. In the ITG of ND brains, a very low percentage of sPLA_2_-IIA-positive astrocytes is present in the thioflavin S-positive plaques. These data suggest that the induction of sPLA_2_-IIA protein in astrocytes could result from their interaction with Aβ and other inflammatory stimuli. This notion is supported by data obtained from experiments using astrocyte cultures derived from post-mortem human brains. Since the IL-1β signaling pathway is considered a key pathway for induction of pro-inflammatory molecules in brain [46], it is possible that a progressive elevation of IL-1β in AD brain could lead to persistent upregulation of inflammatory proteins including sPLA_2_-IIA in astrocytes [47]. Results from astrocyte cultures showed significant induction of sPLA_2_-IIA mRNA by IL-1β or by Aβ alone. These results are in agreement with our previous studies with rat astrocytes [39,48]. Because IL-1β secreted by activated microglia is involved in initiating astrocyte activation and inflammatory cascade [49], its ability to induce sPLA_2_-IIA mRNA in astrocytes suggests that sPLA_2_-IIA upregulation could be engaged in early inflammatory events resulting from astrocyte activation. Taken together, these results are in agreement with the ability of pro-inflammatory cytokines and Aβ to mediate inflammatory responses in astrocytes including the induction of sPLA_2_-IIA.

The apparent lack of sPLA_2_-IIA immunoreactivity in microglial cells seems to be in agreement with our earlier study with a rat stroke model in which up-regulation of sPLA_2_-IIA immunoreactivity was observed primarily in reactive astrocytes but not in microglia [34]. Wang et al. [50] also demonstrated the ability of lipopolysaccharide (LPS) to stimulate and release sPLA2-IIA from astrocytes but not from microglial cells. Results in this study also show immunoreactivity of sPLA_2_-IIA in hippocampal neurons with intensity and staining patterns that are different from those in astrocytes. Since this staining pattern appears in all neurons in both ND and AD samples, more studies are needed to characterize this immunoreactivity. sPLA_2_-IIA immunoreactivity has also been reported in neurons from other brain regions, including Purkinje neurons of rat cerebellum [51]. Aside from sPLA_2_-IIA, other types of sPLA_2 _with similar structure, e.g., groups 1B, IIE, V and X, are present in distinct brain regions [52,53]. Consequently, the functional role of different sPLA_2 _in neurons and glia, and the specific subtypes induced in response to injury, remain an important area to be further explored.

Secretory PLA_2_-IIA has been regarded as an inflammatory protein in the periphery and is upregulated in a number of cardiovascular diseases [25,29,54]. The physiological consequences of inflammatory factors released from glial cells and their ability to damage neurons have been a topic of intense investigation. Our earlier study with astrocytes has demonstrated a role for sPLA_2_-IIA induced by pro-inflammatory cytokines in the production of prostaglandins [39]. Other studies have also shown that secreted sPLA_2_-IIA can perturb cellular membranes, especially those undergoing apoptosis [55-57]. In PC12 cells, lysophospholipids produced by sPLA_2_-IIA were shown to alter neurite outgrowth [58]. Furthermore, sPLA_2 _from bee venom was shown to modulate the activities of ionotropic glutamate receptors and Ca^2+ ^channels, resulting in neuronal excitotoxicity and apoptosis [59,60]. Due to the possible damaging effects of sPLA_2_-IIA on neuronal function, there is strong rationale to develop specific inhibitors for this enzyme [35]. CHEC-9, a peptide inhibitor of sPLA_2_-IIA, was shown to ameliorate PLA_2_-directed inflammation in both acute and chronic neurodegenerative disease models [36]. Our data demonstrating sPLA_2_-IIA as a new inflammatory factor for AD may further facilitate the development of novel therapeutics to retard the progression of this disease.

## Conclusion

This study demonstrates for the first time an increase in protein expression of sPLA_2_-IIA in GFAP-positive astrocytes in AD brains as compared to ND brains. The ability of pro-inflammatory cytokines and Aβ_1–42 _to induce sPLA_2_-IIA mRNA in astrocytes further supports a possible role for sPLA_2_-IIA in the inflammatory responses in AD.

## Abbreviations

AA, arachidonic acid; Aβ, amyloid beta; AD, Alzheimer's disease; cPLA_2_, cytosolic PLA_2_; DAB, diaminobenzidine; DG, dentate gyrus; DMEM, Dulbecco's Modified Eagle Medium; FBS, fetal bovine serum; IFNγ, interferon-γ ; IL-1β, interleukin-1β; ITG, inferior temporal gyrus; GFAP, glial fibrillary acidic protein; ND, non-demented; PBS, phosphate-buffered saline; PCR, polymerase chain reaction; PLA_2_, phospholipase A_2_; sPLA_2_, secretory phospholipase A_2_.

## Competing interests

The author(s) declare that they have no competing interests.

## Authors' contributions

GSDM, LL and DGW acquired samples, performed all of the immunohistochemical studies and PCR analyses of sPLA_2_-IIA mRNA expression in human brains and cultured astrocytes, and edited the manuscript. MDJ, AYS, AS and GYS participated in the design and coordination of the studies and helped to draft the manuscript. GYS, LL, and DGW provided the funding for the project. All authors read and approved the final manuscript.
